# Contents analysis of thyroid cancer-related information uploaded to YouTube by physicians in Korea: endorsing thyroid cancer screening, potentially leading to overdiagnosis

**DOI:** 10.1186/s12889-024-18403-2

**Published:** 2024-04-02

**Authors:** EunKyo Kang, HyoRim Ju, Soojeong Kim, Juyoung Choi

**Affiliations:** 1 National Cancer Control Institute, National Cancer Center, 323 Ilsan-ro, Ilsandong-gu, 10408 Goyang- si, Gyeonggi-do Korea; 2 Department of Family Medicine, National Cancer Center, Goyang, Korea; 3 Department of Family Medicine, Dankook University Hospital, Cheonan, Chungcheongnam-do Korea; 4 Department of Biomedical Science, Seoul National University College of Medicine, Seoul, Korea

**Keywords:** Thyroid cancer screening, Overdiagnosis, Sentiment analysis, YouTube

## Abstract

**Background:**

Thyroid cancer overdiagnosis is a major public health issue in South Korea, which has the highest incidence rate. The accessibility of information through the Internet, particularly on YouTube, could potentially impact excessive screening. This study aimed to analyze the content of thyroid cancer-related YouTube videos, particularly those from 2016 onwards, to evaluate the potential spread of misinformation.

**Methods:**

A total of 326 videos for analysis were collected using a video search protocol with the keyword “thyroid cancer” on YouTube. This study classified the selected YouTube videos as either provided by medical professionals or not and used topic clustering with LDA (latent dirichlet allocation), sentiment analysis with KoBERT (Korean bidirectional encoder representations from transformers), and reliability evaluation to analyze the content. The proportion of mentions of poor prognosis for thyroid cancer and the categorization of advertising content was also analyzed.

**Results:**

Videos by medical professionals were categorized into 7 topics, with “Thyroid cancer is not a ‘Good cancer’” being the most common. The number of videos opposing excessive thyroid cancer screening decreased gradually yearly. Videos advocating screening received more favorable comments from viewers than videos opposing excessive thyroid cancer screening. Patient experience videos were categorized into 6 topics, with the “Treatment process and after-treatment” being the most common.

**Conclusion:**

This study found that a significant proportion of videos uploaded by medical professionals on thyroid cancer endorse the practice, potentially leading to excessive treatments. The study highlights the need for medical professionals to provide high-quality and unbiased information on social media platforms to prevent the spread of medical misinformation and the need for criteria to judge the content and quality of online health information.

**Supplementary Information:**

The online version contains supplementary material available at 10.1186/s12889-024-18403-2.

## Background

Overdiagnosis of thyroid cancer is a global public health issue, especially in South Korea [1–4]. The incidence rate of thyroid cancer in South Korea in 2020 was 56.8%, and the 5-year survival rate is 100%. After the incidence rate of thyroid cancer exceeded 80% in the early 2010s, awareness of the issue of overdiagnosis spread and the establishment of national screening guidelines for thyroid cancer in 2015 [5, 6], there was a temporary decreasing incidence of thyroid cancer until the mid-2010s before it steadily increased again until 2020 [7], with a particularly noticeable increase in the incidence among the younger population. It is important to assess the reason for the increase in excessive screening since 2015 because of the harms of overdiagnosis and consequent overtreatment [8]. 

The decision for screening tests is typically based on the examinee’s consideration of their own health rather than medical screening guidelines [9–11], so the examinee’s understanding of the relevant disease greatly impacts the screening test [9]. The change in accessibility of information could lead to changes in the perception of diseases and may affect excessive screening [11, 12]. South Korea is a country where the accessibility of information through the Internet has rapidly increased [13, 14]. Since the late 2000s, Internet usage in South Korea has rapidly increased, and as of 2021, South Korea is one of the most well-connected countries in the world, with 93% of its population using the Internet [13]. In South Korea, free public Wi-Fi is available in subways and public spaces, and there are various unlimited data plan options [15], which are key factors in improving internet accessibility and affordability. YouTube is the most popular video platform in terms of average monthly usage in South Korea [16], and since the late 2010s, YouTube has rapidly grown. It may have played a crucial role in providing information on medicine and shaping the Korean perception of diseases [17]. 

However, there are concerns about the reliability and quality of online information [18, 19]. In particular, YouTube does not validate the accuracy of the information, and viewers can show blind trust in the words of online medical professionals [20–22]. Content analysis is one way to explore the accuracy of online medical information available on YouTube [23]. Monitoring the reliability and quality of online medical information is most important because incorrect information can cause physical or irreversible damage, and in screening tests, it can lead to the overuse of unnecessary tests or delay of necessary examinations [24, 25]. Furthermore, false beliefs or misunderstandings spread through YouTube could spread distrust of the authorities, cause confusion, and increase public anxiety [26–28]. 

In South Korea, there has been a surge in YouTube videos on medical information released by doctors. Some doctors conduct personal broadcasts or gather to produce broadcast content. Some produce content with the hospital’s support; YouTube has recently attracted attention from medical communication and education [29]. Traditionally, the medical information provided in written form has been criticized for being inaccessible, and this accessibility problem is affecting doctor−patient decision-making [30, 31]. Conversely, YouTube videos can contain multimedia elements, such as graphics, animation, and voice narration, which can improve understanding. Therefore, YouTube can become a user-friendly tool for educating the public on health-related topics.

The increasing incidence rate of thyroid cancer is a major public health issue, especially in South Korea, which has the highest incidence rate. One of the factors of this increase might be the effect of of the thyroid cancer misinformation on the internet platfoem [25]. Therefore, in this study, we aim to analyze the main content of thyroid cancer-related YouTube videos, particularly those from 2016 onwards, and the response of viewers. In addition, through topic-specific analysis, we will conduct a review sentiment analysis and evaluate the audience’s response to videos that recommend exams and emphasize that thyroid cancer is not benign cancer. This will allow us to assess the situation in which misinformation is provided that could affect thyroid cancer screening.

## Methods

### Video search protocol

Since the search protocol through a keyword has been validated in previous studies [32], we searched using the keyword “thyroid cancer” or “thyroidcancer” (there is no spacing between “thyroid” and “cancer” in Korean) on May 1, 2022. The search was performed using a cache-clearing web browser composed of the latest version of Google Chrome in private mode with all available updates. The search results were sorted by “view count” in descending order, and videos with more than 1,000 views and “thyroid cancer” as a main topic were selected. To collect data consistently, we gathered the hyperlink and the view count, titles, and uploaders of the selected video in a spreadsheet. Two reviewers independently reviewed and analyzed all the videos. The exclusion criteria were as follows: duplicate videos; videos without audio and/or video information; videos with a length of less than 1 min; and videos unrelated to thyroid cancer.

A total of 452 videos with over 1,000 views were collected; 103 duplicate videos were excluded, such as videos with the same content but different uploaders. Additionally, 3 videos without sound and 6 videos under 1 min were excluded based on the exclusion criteria. Finally, 14 videos not mainly about thyroid cancer were also excluded. Thus, a total of 326 videos were included for analysis (Supplement Fig. 1). This study was a study that analyzed the contents including YouTube videos, and it was not targeting humans, and there were no expected harms or side effects; therefore, this study did not need ethical approval. Patients or the public were not involved in the design, or conduct, or reporting, or dissemination plans of our research.

### Classification of video providers

The videos were classified as those provided by medical experts or medical institutions (medical professionals) and those that were not. If a government agency or news media provided the video, it was coded as a video provided by a medical expert if it included the opinions of medical experts. Medical professionals were defined as doctors, nurses, and pharmacists, but in actual analysis, all medical experts included in the videos were doctors; because in Korea, there has been a surge in YouTube videos on medical information released by doctors. If the video was uploaded by an individual who is not a medical expert, it was only coded as a video provided by a medical expert if a medical expert appeared in the video and expressed clear opinions. In other words, videos were categorized as those provided by medical professionals if the entity offering the video’s opinion was a medical professional, regardless of the video’s performers or uploaders.

### Topic clustering

Topic clustering is a method for identifying topics in text data and grouping related text documents using unsupervised learning algorithms [33, 34]. After extracting subtitles from YouTube videos, topic clustering was done with the obtained subtitle text. Several algorithms are commonly used for topic clustering, including K-means, hierarchical clustering, latent dirichlet allocation (LDA), and non-negative matrix factorization [33, 34]. This algorithm is based on the word frequency of each document and measures the similarity between documents to group documents with similar topics into clusters. In this study, we used the probabilistic generative model, LDA, to model each document’s topic distribution and conduct topic clustering. This algorithm calculates each document’s topic distribution and the word distribution of each topic. Considering that the characteristics of the videos may differ, topic clustering was carried out by classifying videos provided by medical professionals and those that were not. The videos provided by medical professionals were categorized into a total of 7 topics, and patient experience videos were categorized into 6 topics. The subtitles of the videos classified by LDA were evaluated again to confirm whether each video contained the contents of the corresponding topic.

### Sentiment analysis and contents analysis

In this study, we used bidirectional encoder representations from transformers (BERT), which has shown the most outstanding performance among deep learning-based language models for sentiment analysis [35]. Google publicly released BERT as a pre-trained model for English documents. KoBERT is a model that was trained on Korean data by SKT Brain and has been shown to outperform the multilingual model mBERT in terms of Korean language performance [36, 37]. KoBERT was developed and trained on data, such as Korean Wikipedia and Korean news [36, 37]. In this study, we conducted sentiment analysis using KoBERT for three categories: positive, negative, and neutral. Depending on the classification, whether there was more positive or negative content in the video was compared and finally coded as positive or negative. If there was the most neutral content, it was classified as neutral. One human coder watched the video and classified the content of the video as positive, negative, or neutral, and compared KoBERT’s classification results with the human coding results. The coding results were 100% consistent.

We analyzed the proportion of mentions of poor prognosis for thyroid cancer. Poor prognosis for thyroid cancer was defined as a direct mention of poor thyroid cancer prognosis, such as “thyroid cancer is fatal cancer,” “the mortality rate of thyroid cancer is high,” and “thyroid cancer is not good cancer.” In addition, videos based on advertising content were categorized into insurance advertisements, alternative medicine, and therapies through food (advertisements for specific foods). This was done to determine which advertising information is provided.

### Reliability evaluation

Videos classified as applicable were further evaluated for their reliability. There currently needs to be a consensus on how to evaluate the reliability of medical information included in videos. Therefore, we reviewed evaluation tools commonly used in previous research on online consumer health information, such as the Journal of the American Medical Association Scoring (JAMAS) and Global Quality Score (GQS) for website tools and DISCERN for written patient information on treatment choices [38–40]. Considering the evaluation of information on thyroid cancer, the reliability of the videos was assessed using a modified DISCERN score. It is the first standardized Consumer Health Information Quality Index that can be used as an essential evaluation tool not only for healthcare professionals but also for patients and the general public in evaluating health information. The maximum total score of DISCERN is 5 points [40]. The evaluation was conducted by two doctors with over 5 years of experience in general hospitals, including counseling for cancer screening, including thyroid cancer. The average score of independent DISCERN scores by the two physicians was used to confirm the reliability of video evaluations. The consistency between the two coders was 0.842 (The overall Cronbach’s alpha is 0.842). Two researchers independently evaluated each video, and the average DISCERN score for the videos was 3.72 points.

## Results

### YouTube video characteristics

Table 1 presents the characteristics of the total 326 YouTube videos analyzed. YouTube videos included in the analysis were divided into videos created by medical professionals (*N* = 169) and non-experts (*N* = 157). Table 1 shows the distribution of videos based on the year of creation, where the total number of videos created before 2015 was 20 (6.1%), and most videos were created in 2021 (97 videos, or 29.8%). There is a difference in the ratio of uploader every year, but while there were more videos uploaded by medical staff from 2015 to 2020, videos by non-professionals tended to account for a larger percentage from 2021. The average length of a YouTube video was 12.7 ± 0.9 min. The average length of videos produced by medical professionals was significantly longer than those created by non-experts (15.8 ± 1.4 vs. 9.3 ± 1.1 min, *p* = 0.002). In other words, it was confirmed that videos provided by medical staff provided more information on average. Videos created by medical.

professionals had a higher average number of views (31,270.9 ± 6,198.8) but a lower average number of comments (48.4 ± 12.9) than videos created by non-professionals. Many video recommendations were made by medical professionals (470.3 ± 103.2); It was confirmed that significantly more viewers watched the videos provided by medical staff, recommended them more, but left relatively fewer comments. Videos uploaded by videos, advertisement videos, and other videos. Among them, patient experience videos accounted for almost half of the total (45.2%).

**Table 1:** The characteristics of YouTube videos

		Total (*N* = 326)	By medical professionals (*N* = 169)	By non-professionals (*N* = 157)	*P*-value
**Year, N (%)**	Before 2015	20 (6.1)	11 (55.0)	9 (45.0)	< 0.001
	2016	9 (2.8)	9 (100.0)	0 (0.0)	
	2017	6 (1.8)	4 (66.7)	2 (33.3)	
	2018	13 (4.0)	8 (61.5)	5 (38.5)	
	2019	41 (12.6)	21 (51.2)	20 (48.8)	
	2020	96 (29.5)	53 (55.2)	43 (44.8)	
	2021	97 (29.8)	47 (48.5)	50 (51.5)	
	2022	44 (13.5)	16 (36.4)	28 (63.6)	
**Length of video**, Mean ± SD* (min)		12.7 ± 0.9	15.8 ± 1.4	9.3 ± 1.1	0.002
**Average** **number** **of**	Views	28,046.0 ± 4,769.6	31,270.9 ± 6,198.8	24,574.6 ± 7,329.3	< 0.001
	Comments	37.7 ± 6.8	27.6 ± 5.1	48.4 ± 12.9	< 0.001
	Recommend	379.5 ± 68.2	470.3 ± 103.2	281.8 ± 87.4	< 0.001
**Uploaders**	Patients			71 (45.2)	
	Advertisement			31 (19.7)	
	Others			55 (35.1)	

*SD: Standard deviation

### Topic clustering of videos by medical professionals and sentiment analysis of comments

**Fig. 1:**
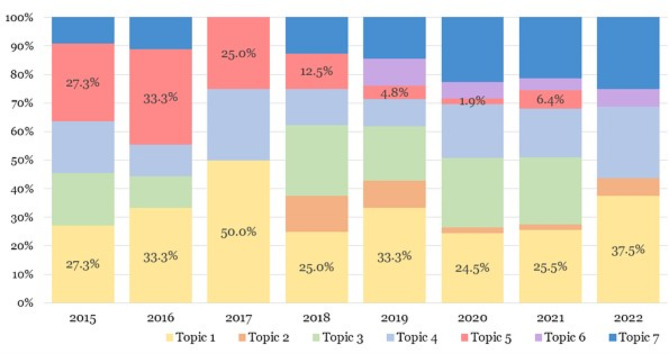
Proportion of topics in YouTube videos by year (in which they were released)

The videos provided by medical professionals were categorized into a total of 7 topics (Table 2). The topic that accounted for the highest percentage was “Thyroid cancer is not a ‘Good cancer,’” which accounted for 28.4% of all videos. The next most common topics were “Symptoms and diagnosis of thyroid cancer” (19.5%) and “Management of thyroid cancer patients” (18.9%). An analysis was conducted not only to determine the frequency of videos stating that thyroid cancer is not a “good cancer”, but to ascertain the proportion of videos across different topics mentioning that thyroid cancer is not a “good cancer” (i.e., having a poor prognosis). Among all videos by medical professionals analyzed, approximately 68.5% mentioned that thyroid cancer has a poor prognosis, which accounts for nearly two-thirds of the total videos. The video that peoples most viewed was “Symptoms and diagnosis of thyroid cancer,” with an average view count of 75,885.6 ± 23,950.3. However, in terms of recommendations, “Management of thyroid cancer patients” was the most recommended. To evaluate peoples’ reactions to the videos, sentiment analysis was conducted on the comments (Fig. 1). The topic with the most positive comments was “Thyroid cancer is not a ‘Good cancer,’” which had relatively more positive reactions urging people to get screened for thyroid cancer. Conversely, videos about thyroid cancer overdiagnosis had more negative than positive comments. In the year-by-year analysis of the topics covered in videos provided by physicians from 2015 to 2022, Topic 1 (“Thyroid cancer is not a ‘Good cancer’”) remained at a similar level, while Topic 5 (“Overdiagnosis of thyroid cancer”) showed a gradual decrease.

**Table 2:** Topic clustering of videos by medical professionals and sentiment analysis of comments

		*N* (%)	Mentioned poor prognosis (*N* (%))	Average number of views (Mean ± SD)	Average number of recommend (Mean ± SD)	Comments (included in the analysis*)	Comments (%)		
							Positive	Neutral	Negative
**Topic 1**	**Thyroid cancer is not a** **“Good cancer”**	48 (28.4)	48 (100.0)	12,785.9 ± 2,058.8	193.8 ± 35.6	819 (623)	70.1	14.8	15.1
**Topic 2**	**Epidemiology and causes of** **thyroid cancer**	6 (3.6)	4 (66.7)	4,689.5 ± 2,861.4	59.2 ± 38.1	37 (32)	46.9	37.5	15.6
**Topic 3**	**Symptoms and diagnosis of** **thyroid cancer**	33 (19.5)	27 (81.8)	75,885.6 ± 23,950.3	902.7 ± 313.2	1,677 (1,352)	61.1	18.5	20.4
**Topic 4**	**Treatment of thyroid cancer**	28 (16.6)	22 (78.6)	12,224.1 ± 2,363.3	247.5 ± 63.4	683 (571)	60.9	15.8	23.3
**Topic 5**	**Overdiagnosis of** **thyroid cancer**	14 (8.3)	0 (0.0)	9,684.0 ± 2,167.8	108.5 ± 39.4	97 (81)	30.9	28.4	40.7
**Topic 6**	**Prognosis of thyroid cancer**	8 (4.7)	2 (25.0)	5,067.6 ± 3,741.7	74.1 ± 56.9	52 (38)	34.2	39.5	26.3
**Topic 7**	**Management of** **thyroid cancer patients**	32 (18.9)	12 (37.5)	50,634.3 ± 18,758.7	968.7 ± 412.7	1,277 (964)	68.7	14.9	16.4

### Topic clustering of videos about patient experience and sentiment analysis of comments

Patient experience videos were categorized into 6 topics (Table 3). The topic with the highest proportion was Topic 3 (“Treatment process and after-treatment of thyroid cancer”), followed by Topic 5 (“Thyroid cancer sequelae/complications”) and Topic 6 (“The story of overcoming thyroid cancer”). In Topic 3, many videos included the importance of receiving thyroid cancer screenings and information about the treatment. This topic showed predominantly negative sentiments toward thyroid cancer. However, compared to the videos provided by medical staff, the rate of mention of the poor prognosis of thyroid cancer was significantly lower in the videos provided by non-professionals (68.5% vs. 39.4%, *p* < 0.001).

**Table 3:** Topic clustering of videos about patient’s experience and sentiment analysis of comments (*N* = 71)

		*N* (%)	Mentioned poor prognosis (*N* (%))	Average number of views (Mean ± SD)	Average number of recommend (Mean ± SD)	Comments (included in the analysis)	Comments (%)		
							Positive	Neutral	Negative
**Topic 1**	**Symptoms and detection** **of thyroid cancer**	6 (8.5)	0 (0.0)	6,273.5 ± 2,826.7	73.5 ± 21.8	96 (85)	51.8	8.2	40.0
**Topic 2**	**Diagnosis process of** **thyroid cancer**	5 (7.0)	0 (0.0)	84,926.6 ± 46,847.6	1,085.8 ± 622.0	1,339 (1,185)	57.9	9.6	32.5
**Topic 3**	**Treatment process and** **aftertreatment of** **thyroid cancer**	28 (39.4)	14(50.0)	61,179.9 ± 35,843.2	589.4 ± 387.5	3,553 (2,977)	63.6	19.8	16.7
**Topic 4**	**Management after** **thyroid cancer treatment**	9 (12.7)	2(22.2)	34,412.3 ± 28,568.0	813.1 ± 712.3	774 (672)	74.3	10.6	15.2
**Topic 5**	**Thyroid cancer sequelae/** **complications**	13 (18.3)	10 (76.9)	13,756.4 ± 4,343.1	160.1 ± 36.6	546 (470)	56.6	20.4	23.0
**Topic 6**	**The story of overcoming** **thyroid cancer**	10 (14.1)	2(20.0)	1,863.6 ± 579.7	49.9 ± 13.5	152 (131)	82.4	9.2	8.4

In Topic 3 and Topic 5, there were many cases of direct mention of poor prognosis for thyroid cancer (Supplement Fig. 2). These included mentions of the complex treatment process for thyroid cancer, the importance of early detection, and their impression that it is not a “good” cancer. There were also mentions of complications and after-effects that can occur with thyroid cancer, emphasizing the need for screening.

### Classification of videos that included advertisements and sentiment analysis of comments

The videos that included advertisements were broadly classified into 3 categories, of which insurance advertisements accounted for more than half (61.3%) (Table 4). These videos advertised the possibility of claiming insurance for thyroid cancer and whether thyroid cancer screenings were covered by insurance. Many of the advertisements were found to include content that encourages screening.

**Table 4:** Classification of videos including advertisements and sentiment analysis of comments

	*N* (%)	Average number of views (Mean ± SD)	Average number of recommend (Mean ± SD)	Comments (included in the analysis)	Comments (%)		
					Positive	Neutral	Negative
**Advertisement of** **insurance** **(company)**	19 (61.3)	1,983.2 ± 278.8	33.7 ± 8.3	171 (113)	69.0	6.2	24.8
**Advertisement of** **food therapies**	7 (22.6)	31,767.7 ± 14,849.9	622.0 ± 300.1	203 (184)	83.7	6.5	9.8
**Advertisement of** **alternative** **medicine/nursing** **facilities**	5 (16.1)	1,258.0 ± 100.2	44.6 ± 17.3	66 (58)	86.2	8.6	5.2

## Discussion

Thyroid cancer, which, since 2014, had been showing a decreasing trend in Korea after the problem of overdiagnosis was raised [41], rebounded after 5 years to become the top cancer in 2019. However, despite the increase in new cases of thyroid cancer, the mortality rate has not changed [7]. Thyroid cancer remained the most common cancer in 2020. However, unlike other cancers, thyroid cancer has a 5-year relative survival rate of 100%, indicating a favorable prognosis [42]. Nevertheless, thyroid cancer screenings are being overused by private institutions in Korea [43]. Given that unnecessary screenings could easily lead to excessive treatments, there is an increasing need to investigate the reasons for the increasing number of thyroid cancer screenings.

In this study, we found that a significant proportion of thyroid cancer-related videos uploaded by medical professionals endorse the practice, and the number of videos opposing excessive thyroid cancer screening is gradually decreasing yearly. In addition, it was confirmed that the proportion of videos that mentioned the poor prognosis of thyroid cancer and showed a supportive stance on thyroid cancer screening was significantly higher in the videos provided by medical staff compared to the videos provided by non-professionals. The sentiment of comments from viewers and for videos based on patient experiences was more favorable for videos advocating thyroid cancer screening than those opposing the screening. In addition, we found that advertisements by insurance companies advocating thyroid cancer screening had the highest proportion. The changing topics of these videos may be related to the increased incidence of thyroid cancer in Korea. The fact that the topics of videos provided by medical professionals are changing suggests that these videos have the potential to positively influence the perceptions of screening by viewers who search for and accept such information.

Access to online health information is increasing worldwide, including in Korea [14, 44]. As a result of this increased accessibility, consumers may be exposed to medical information without barriers; and are more likely to believe that information is accurate when it is uploaded by a reliable source [18, 45]. For this reason, YouTube videos uploaded by medical experts generally receive a high number of positive comments and can be viewed with a high level of trust. In this study, the number of videos uploaded by medical professionals criticizing excessive thyroid cancer screening decreased since 2015, while the number of videos advocating thyroid cancer screening increased. This attitude of medical professionals may create a public opinion that thyroid cancer screening is necessary, which may lead to excessive screening; therefore, medical professionals on social media need to share high-quality information.

In this study, we examined the relationship between information provided by medical professionals and the increase in thyroid cancer screening, a public health phenomenon. We found that comments from viewers on topics advocating thyroid cancer screening were more positive than those on topics opposing the screening. This suggests that viewers are more likely to believe in false health information that leads to overdiagnosis, if it could offset their fear. Previous research has reported a significant negative correlation between scientific quality and viewer engagement among cancer information videos available on YouTube [46, 47]. Users are more likely to watch lower-quality or biased videos than high-quality ones. However, this suggests that medical misinformation could spread quickly because most social media platforms’ algorithms push content based on more views or engagement [48, 49]. Given the nature of social media platforms, where videos advocating thyroid cancer screening that elicit positive responses from viewers may be more exposed, it is evident that basic verification of medical information in social media is necessary.

YouTube provides a variety of reliable sources for cancer-related news and information, including medical experts, non-profit organizations, universities, and hospital-based websites. In this study, we analyzed the video content provided by experts and non-experts on YouTube. The results of this study revealed that the information presented in YouTube videos often did not align with the opinions of expert groups, potentially leading patients to accept and follow incorrect information. One of the most concerning findings of this study was that among YouTube videos about thyroid cancer, more videos were provided by medical staff advocated for screening and the positive response from viewers in the comments section of videos advocating for thyroid cancer screening. As a result, the role of medical professionals in social media platforms where information providers and consumers coexist should be to monitor and flag incorrect information collectively to prevent public health phenomena, such as excessive screening. Setting verification standards focused on health and science information accounts is essential to prevent misinformation and help users better distinguish between accurate and potentially misleading information [50]. 

The limitation of this study is that only YouTube was analyzed as an information-providing platform. However, given that YouTube is the most active social media platform among medical professionals to provide information online, analyzing this platform may be the most suitable way to understand the flow of information changes. Additionally, in this study, only currently searchable videos were included after the establishment of the thyroid screening guidelines in 2015, which means that deleted videos were excluded from the analysis. Most of the deleted videos are likely to be unpopular or contain misinformation, so their exclusion does not pose a problem for the results of this study. Based on the results of this study, we have confirmed the need for criteria for judging the content and quality of online information, especially when healthy consumers make decisions on selective tests, such as screening, based on the online information they receive. We also confirmed the need for self-filtering in the information provided by medical professionals to prevent excessive thyroid cancer screenings in Korea. In the future, there is a need for YouTube content to shift toward preventing excessive thyroid cancer screenings in order to lower the screening rate.

## Conclusion

Thyroid cancer overdiagnosis is a significant public health issue in South Korea, with the highest incidence rate worldwide. With the increased accessibility of information through the internet, particularly on YouTube, there is potential for the spread of medical misinformation and the promotion of excessive screening practices. Our study aimed to analyze the content of thyroid cancer-related YouTube videos, specifically those from 2016 onwards, to evaluate the potential spread of misinformation. Our findings revealed that a significant proportion of videos uploaded by medical professionals on thyroid cancer endorse the practice, potentially leading to excessive treatments. Moreover, we found that the sentiment of comments from viewers was more favorable for videos advocating thyroid cancer screening than those opposing the screening.

This study highlights the need for medical professionals to provide high-quality and unbiased information on social media platforms to prevent the spread of medical misinformation. Furthermore, in the case of health information, more accurate information is needed to prevent incorrect medical practices such as excessive screening. Therefore, there is a need for evaluation criteria for health information on social media and ongoing monitoring through those criteria. Additionally, to prevent misinformation and help users better distinguish between accurate and potentially misleading information, it is essential to establish a standard format for indicating evidence regarding the content when uploading it to health and science information accounts. For this purpose, the establishment of standards for medical and scientific evidence labeling for health information is crucial. This should be considered as a key strategy at the government level in the National Cancer Control Plan (NCCP), particularly for improving cancer awareness in the advancement of public health. Our findings could contribute to public health policymaking to improve thyroid cancer screening practices in South Korea.

### Electronic supplementary material

Below is the link to the electronic supplementary material.

Supplementary Material 1

## Data Availability

The datasets generated during and/or analyzed during the current study are available from the corresponding author on reasonable request.
