# Supplementary material for: Contents analysis of thyroid cancer-related information uploaded to YouTube by physicians in Korea: endorsing thyroid cancer screening, potentially leading to overdiagnosis
Source: BMC Public Health. 2024 Apr 2;24:942. doi: 10.1186/s12889-024-18403-2 (PMC10985908; doi:10.1186/s12889-024-18403-2)
Supplement: Supplementary file 1 — Supplementary Material 1 [file 12889_2024_18403_MOESM1_ESM.docx]

**Supplement figure 1.** Flow diagram for the video being analyzed

**
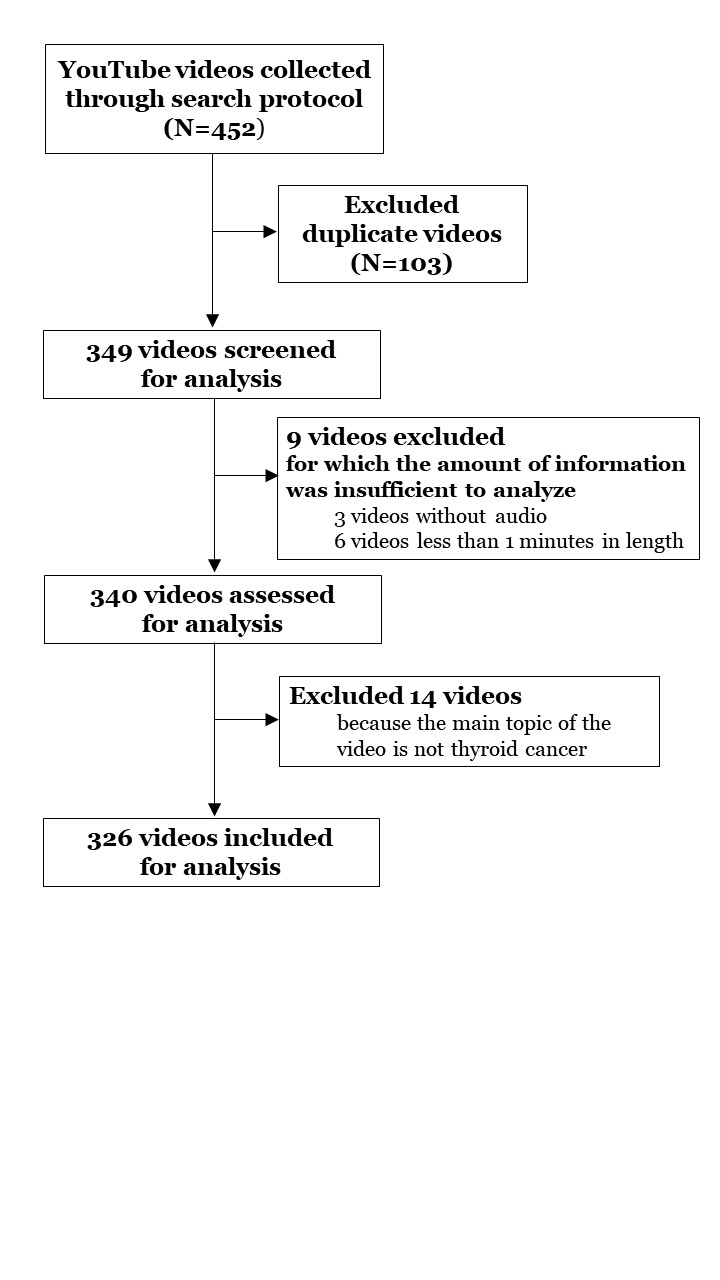
**

**Supplement figure 2.** Ratio of mentions of poor prognosis by topic in videos about patient’s experience

**
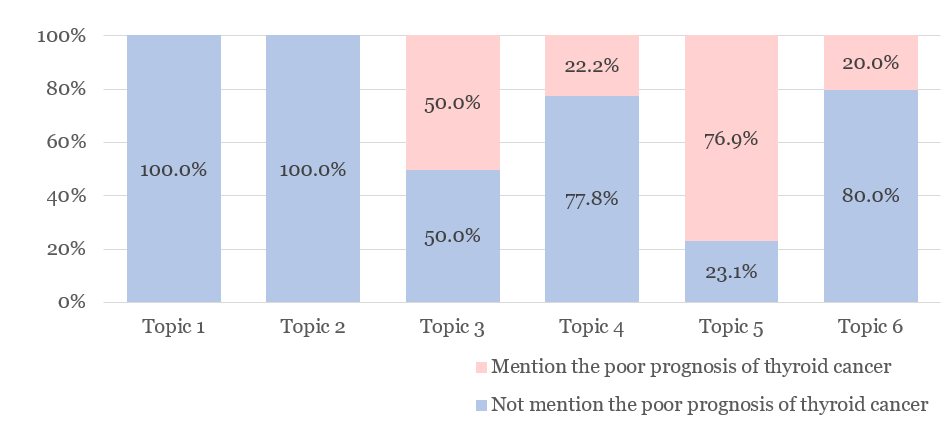
**
